# Nomogram-based parameters to predict overall survival in a real-world advanced cancer population undergoing palliative care

**DOI:** 10.1186/s12904-019-0432-7

**Published:** 2019-06-05

**Authors:** Weiwei Zhao, Zhiyong He, Yintao Li, Huixun Jia, Menglei Chen, Xiaoli Gu, Minghui Liu, Zhe Zhang, Zhenyu Wu, Wenwu Cheng

**Affiliations:** 1 Department of Integrated Therapy, Fudan University Shanghai Cancer Center, Shanghai, China; 2 Department of Oncology, Shanghai Medical College, Fudan University, Shanghai, China; 3 Department of Anesthesiology, Fudan University Shanghai Cancer Center, Shanghai, China; 4 Department of Oncology, Shandong Cancer Hospital, Shandong Academy of Medical Sciences, Jinan, China; 5 Department of Biostatistics, School of Public Health, Key Laboratory of Public Health Safety and Collaborative Innovation Center of Social Risks Governance in Health, Fudan University, Shanghai, China

**Keywords:** Nomogram, Palliative care, Survival prediction, Advanced cancer

## Abstract

**Background:**

Although palliative care has been accepted throughout the cancer trajectory, accurate survival prediction for advanced cancer patients is still a challenge. The aim of this study is to identify pre-palliative care predictors and develop a prognostic nomogram for overall survival (OS) in mixed advanced cancer patients.

**Methods:**

A total of 378 consecutive advanced cancer patients were retrospectively recruited from July 2013 to October 2015 in one palliative care unit in China. Twenty-three clinical and laboratory characters were collected for analysis. Prognostic factors were identified to construct a nomogram in a training cohort (*n* = 247) and validated in a testing cohort (*n* = 131) from the setting.

**Results:**

The median survival time was 48.0 (95% CI: 38.1–57.9) days for the training cohort and 52.0 (95% CI: 34.6–69.3) days for the validation cohort. Among pre-palliative care factors, sex, age, tumor stage, Karnofsky performance status, neutrophil count, hemoglobin, lactate dehydrogenase, albumin, uric acid, and cystatin-C were identified as independent prognostic factors for OS. Based on the 10 factors, an easily obtained nomogram predicting 90-day probability of mortality was developed. The predictive nomogram had good discrimination and calibration, with a high C-index of 0.76 (95% CI: 0.73–0.80) in the development set. The strong discriminative ability was externally conformed in the validation cohort with a C-index of 0.75.

**Conclusions:**

A validated prognostic nomogram has been developed to quantify the risk of mortality for advanced cancer patients undergoing palliative care. This tool may be useful in optimizing therapeutic approaches and preparing for clinical courses individually.

**Electronic supplementary material:**

The online version of this article (10.1186/s12904-019-0432-7) contains supplementary material, which is available to authorized users.

## Background

Cancer constitutes an enormous burden on society worldwide [1]. Since 2010, cancer has become the leading cause of death in China, and the incidence and mortality continue to grow [2, 3]. Synchronously, palliative care has become a growing worldwide issue and has been integrated into the trajectory of standard cancer care [4, 5]. In the palliative care unit, survival prediction is required irrespective of the underlying types of cancer.

Prognostication of life expectancy can provide positive significance: preparing patients and families with affair-setting and reasonable care expectations; assisting physicians with clinical decision-making; providing researchers with clinical trial design and follow-up tactics; helping governors with policy planning [6–8]. However, it is difficult to predict the overall survival (OS) for mixed advanced cancer patients with limited life expectancy and poor physical states. Under the circumstances, clinicians’ optimistic survival predictions often lead to inappropriate therapeutic or discharge recommendations, as well as delayed referrals to palliative or hospice programs [9, 10]. Although extensive research has been performed to predict the survival outcome of advanced cancer patients, no acknowledged predictive model has been established in the palliative care unit. Thus, accurate and practical prognostic tools must be developed for the efficient prognosis of advanced cancer patients at the bedside.

A nomogram is a useful tool for predicting clinical outcomes via a simple and clear figure [11]. The development of nomograms with methodological superiority would reduce prognostic uncertainty and has been increasingly used for survival prediction in the field of cancer [12]. Unfortunately, nomograms for survival prediction of mixed advanced cancer patients in the palliative care unit are scarce.

In this clinical scenario, we identified the independent prognostic factors pre-palliative care and then developed a novel nomogram with an externally validation to further refine the survival prediction for advanced cancers patients in a real-world palliative care setting.

## Methods

### Patients and study dataset

Patients treated at the palliative care unit of Fudan University Shanghai Cancer Center (FUSCC), Shanghai, China were retrospectively reviewed. Between July 2013 and December 2014, 247 consecutively advanced cancer patients (training cohort) were analyzed to identify prognostic factors and create the survival prognostic nomogram. Subsequently, from January 2015 to October 2015, 131 attended advanced cancer patients (validation cohort) were recruited to validate the prognostic scale. The last follow-up date was in February 2016. The primary outcome was OS, which was defined as the period from the date of initial treatment in the palliative care unit of FUSCC to death or the last follow-up (if death was not known). This study was approved by the institutional review board of Fudan University Shanghai Cancer Center. Informed consent was waived because of the retrospective nature of the study. Details of inclusion/exclusion criteria and follow-up procedure (Additional file 1: Figure S1) of the patients are described in the Supplementary Methods.

### Data collection

A total of 23 clinical and laboratory features for each patient were collected from the medical records platform of FUSCC by trained staff using standard data collection and quality-control procedures. Data included demographics (sex, age, body mass index), medical history (hospital stay, concomitant disease), tumor-related factors [primary tumor site (gastrointestinal/ thoracic/ urogenital/ head and neck/ other tumors) and tumor stage], nutritional status, physical status [Karnofsky performance status (KPS) score], and laboratory variables [neutrophil count, lymphocyte count, hemoglobin, platelet count, total bilirubin, alkaline phosphatase, aminoleucine transferase, aspartate aminotransferase, lactate dehydrogenase (LDH), albumin, creatinine, uric acid (UA), cystatin-C, carcinoembryonic antigen], which were performed 1–3 days before the start of palliative care. An unintentional weight loss > 5% in the previous 3 months or a food intake below 75% of the normal requirement in the preceding week were considered to represent abnormal nutritional status according to the ESPEN guidelines for nutrition screening [13]. The presence of comorbidity was defined as self-reported cardiac disease, hypertension, diabetes, or any cerebrovascular disease. All these parameters were collected before initiating palliative care.

### Statistical analysis

Categorical variables were described as totals and frequencies, and differences between the training and validation cohorts were determined using the chi-squared or Fisher’s exact test as appropriate. Continuous variables were described as medians and interquartile ranges (IQR) and compared by the Wilcoxon rank-sum test or Kruskal-Wallis *H* test.

The associations between survival and clinical or laboratory features were evaluated using Cox proportional hazards regression analysis. To determine the strongest predictors in the final model, variables with significant level < 0.05 in univariate analysis were considered in the multivariate models and the backward elimination method was used and a variable was considered for addition to or subtraction from the set of variables based on the Akaike information criterion (AIC). A nomogram for predicting survival was developed based on the significant features in the model. The nomogram performance was composed of two components: discrimination and calibration. The ability of a model to separate subject outcomes is known as discrimination. Discrimination was quantified with the concordance index (C-index), which is similar to the area under the receiver operating characteristic (ROC) curve [14]. The C-index ranges from 0 to 1, where 1 indicates perfect discrimination, 0.5 indicates no better concordance than chance and 0 indicates perfect discordance. A C-index value over 0.75 is usually considered to represent relatively good discrimination. Calibration was performed by comparing the predicted probability of survival versus the actual probability of survival [11], again using 200 bootstrap re-samples to reduce the overfit bias, which would overstate the accuracy of the nomogram. In a well-calibrated plot, the predictions should fall on a 45-degree diagonal line. Calibration of the model was assessed graphically. We validated the nomograms with an external independent validation cohort, and the predictive performance was evaluated by the C-index value and calibrated plot. The decision curve analysis (DCA), a novel method to evaluate prediction models from the perspective of clinical consequences by calculating the net benefit was also performed. Statistical analyses were performed using SAS 9.4 (SAS Institute Inc., Cary, NC) and R software version 3.2.3 (http://www.r-project.org) with the rms package. When the *p*-value was less than 0.05, the difference was considered statistically significant.

## Results

Basic information and clinical characteristics of all 378 consecutive advanced cancer patients (247 patients in the training cohort and 131 patients in the validation cohort) are summarized in Table 1. The median duration of follow-up of the training cohort and validation cohort was 598 days (95% CI: 494–689) and 178 days (95% CI: 139–265), respectively. Most variables were similar between the two cohorts, excluding concomitant disease (*p* = 0.0101) and cystatin-C (*p* = 0.0218). We found that 85.4% (211/247) of the advanced cancer patients in the training cohort and 74.8% (98/131) of the advanced cancer patients in the validation cohort had died by February 2016. The median survival time in the two cohorts was 48.0 days (95% CI: 38.1–57.9) and 52.0 days (95% CI: 34.6–69.3), respectively.

**Table 1:** Characteristics of patients in the training cohort and the validation cohort

Clinicopathological features	Training cohort	Validation cohort	*P* value
No. of patients	247	131	
Sex			
Male	133(53.85%)	76(58.02%)	0.4379
Female	114(46.15%)	55(41.98%)	
Age			
<70y	162(65.59%)	94(71.76%)	0.2222
> = 70y	85(34.41%)	37(28.24%)	
Tumor stage			
III	15(6.07%)	8(6.11%)	0.9895
IV	232(93.93%)	123(93.89%)	
Primary tumor site			
Gastrointestinal tumors	125(50.61%)	73(55.73%)	0.1352
Thoracic cancers	66(26.72%)	20(15.27%)	
Urogenital neoplasms	34(13.77%)	25(19.08%)	
Head and neck neoplasms	10(4.05%)	6(4.58%)	
Other tumors	12(4.86%)	7(5.34%)	
Concomitant disease			
No	138(55.87%)	91(69.47%)	0.0101
Yes	109(44.13%)	40(30.53%)	
Nutritional status			
Normal	71(28.98%)	36(27.69%)	0.7928
Abnormal	174(71.02%)	94(72.31%)	
Hospital stay			
<=14d	131(53.04%)	74(56.49%)	0.5215
>14d	116(46.96%)	57(43.51%)	
KPS	60(10–90)	60(10–90)	0.1192
Body mass index, kg/m^2^	21.10(12.62–35.38)	20.80(13.67–34.05)	0.5285
Total bilirubin, umol/L	11.45(3.2–920)	11.95(2.3–389.3)	0.7913
Alkaline phosphatase, U/L	122.15(39.8–2116.1)	120.85(37.1–1342.6)	0.4955
ALT, U/L	17.85(5–914.2)	19.15(5–1156)	0.9394
AST, U/L	25.25(4.9–2435.5)	28.55(8.6–1041.4)	0.9627
Lactate dehydrogenase, U/L	234(81–2651)	217(85–2101)	0.4242
Cystatin-C, mg/L	1.25(0.65–5.7)	1.135(0.57–4)	0.0218
Creatinine, umol/L	67(26–468)	63(32–406)	0.1013
Platelet count, No. × 10^9^/L	217(10–872)	201.5(26–753)	0.1819
Neutrophil count, No. × 10^9^/L	6.2(1–91.7)	5.55(1.2–96)	0.0535
Lymphocyte count, No. ×10^9^/L	1.1(0.1–32.1)	1.1(0.3–4.2)	0.6950
Hemoglobin, g/L	107(42–169)	109.5(32–171)	0.5162
CEA, ng/ml	4.76(0.32–929.1)	3.83(0.44–975.1)	0.4807
Albumin, g/L	33.6(17.3–48.4)	34.5(13.5–48.4)	0.1863
Uric acid, umol/L	314.5(71.0–1560.0)	323(70–1073)	0.6762

Abbreviation: *KPS* karnofsky performance status score, *CEA* carcinoembryonic antigen, *ALT* Glutamic-pyruvic transaminase, *AST* Glutamic-oxalacetic transaminase

To provide survival estimates in a more useful manner, we undertook the retrospective study with the 23 easily obtained clinical parameters. The results of the multivariate Cox proportional hazards regression analysis are listed in Table 2, including demographics, medical history, tumor-related factors, nutritional status, physical status and laboratory variables. Multivariate analyses of the training cohort demonstrated that sex (adjusted HR = 1.338; 95% CI: 0.990–1.808; *p* = 0.056), age (adjusted HR = 0.603; 95% CI: 0.442–0.822; *p* = 0.004), tumor stage (adjusted HR = 4.104; 95% CI: 1.773–9.495; *p* = 0.001), KPS (adjusted HR = 0.977; 95% CI: 0.969–0.985; *p* < 0.001), neutrophil count (adjusted HR = 1.015; 95% CI: 1.007–1.024; *p* = 0.121), hemoglobin (adjusted HR = 0.999; 95% CI: 0.998–1.000; p < 0.001), LDH (adjusted HR = 1.001; 95% CI: 1.000–1.001; *p* < 0.001), albumin (HR = 1.338; 95% CI: 0.990–1.808; *p* < 0.001), UA (HR = 0.933; 95% CI: 0.904–0.964; *p* = 0.022) and cystatin-C (adjusted HR = 3.171; 95% CI: 2.186–4.600; *p* < 0.001) were independent risk factors for OS (Table 2).

**Table 2:** Significant prognostic factors associated with the OS according to the AIC criterion

	Adjusted HR	95% CI	P value
Sex	1.338	0.99 ~ 1.808	0.056
Age	0.603	0.442 ~ 0.822	0.004
TNM	4.104	1.773 ~ 9.495	0.001
KPS	0.977	0.969 ~ 0.985	< 0.001
LDH	1.001	1.000 ~ 1.001	< 0.001
Cystain-C	3.171	2.186 ~ 4.6	< 0.001
Neutrophile	1.015	1.007 ~ 1.024	0.121
Hemoglobin	0.999	0.998 ~ 1.000	< 0.001
UA	0.933	0.904 ~ 0.964	0.022
Albumin	1.338	0.99 ~ 1.808	< 0.001

Abbreviation: *KPS* karnofsky performance status score, *LDH* lactate dehydrogenase; *UA* Uric acid

**Fig. 1:**
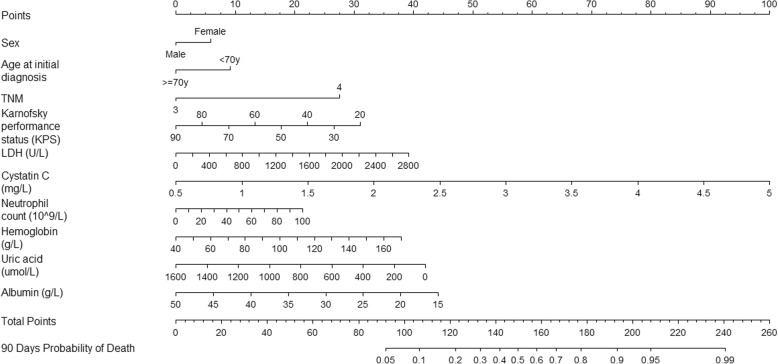
Nomogram of 90 days probability of death (To use the nomogram, an individual patient’s value is located on each variable axis, and a line is drawn upward to determine then number of points recenvied for each variable value. The sum of these numbers is located on the Total Points axis, and a line is drawn downward to the suvival axes to determine the propobality of 90 days death). Abbreviation: KPS, karnofsky performance status score; LDH, Lactate dehydrogenase; UA, Uric acid.

**Fig. 2:**
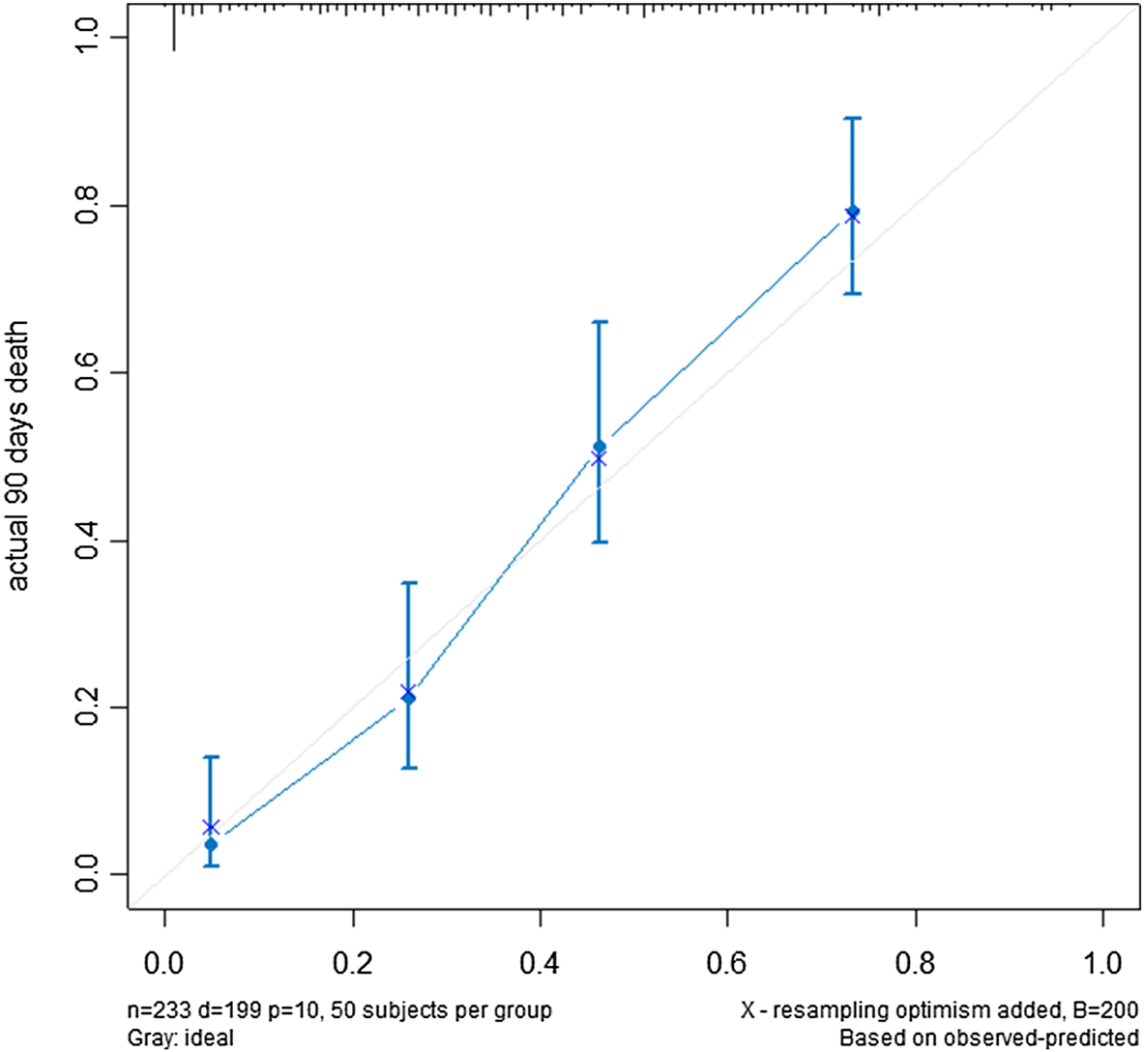
Calibration of the nomogram in the training cohort (*n* = 247). The x-axis represents the nomogram-predicted survival, and the y-axis represents actual survival and 95% CIs measured by Kaplan-Meier analysis

**Fig. 3:**
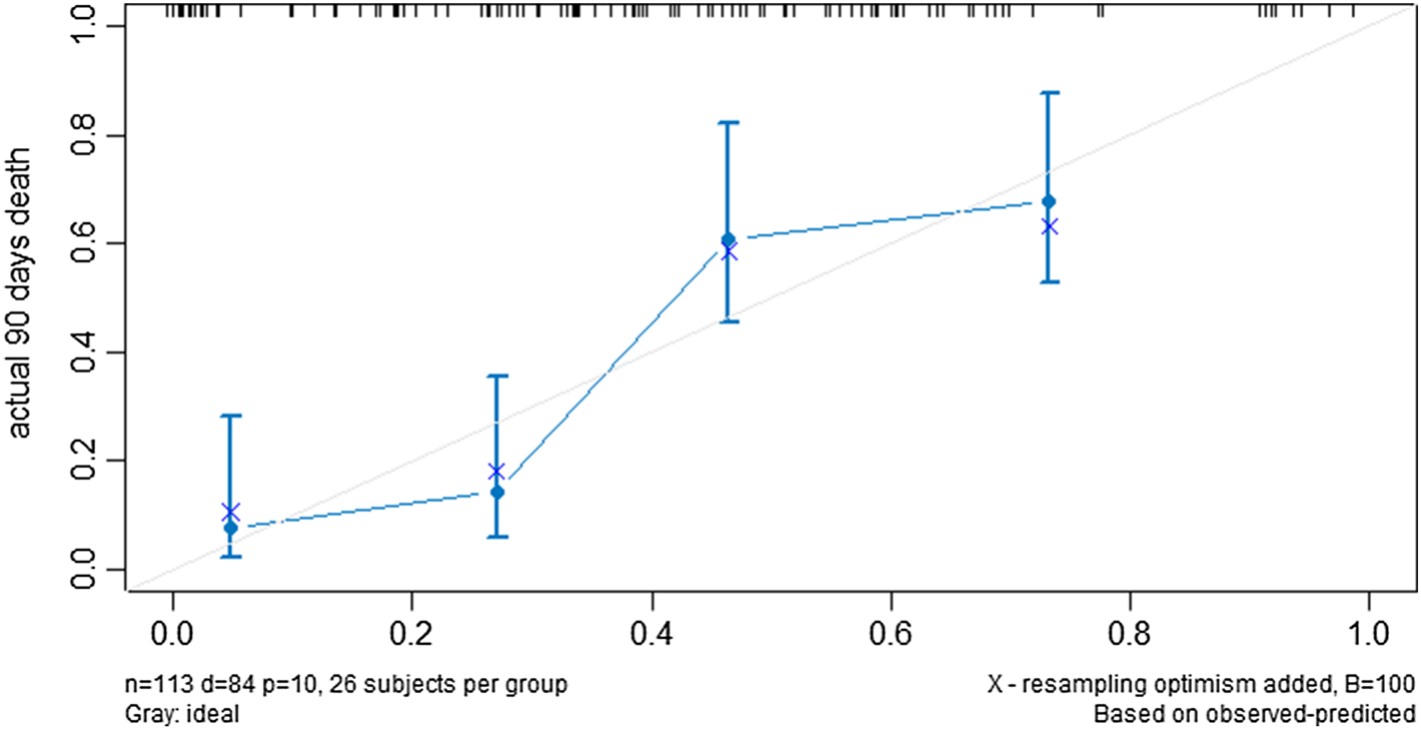
Calibration of the nomogram in the testing cohort (*n* = 131). The x-axis represents the nomogram-predicted survival, and the y-axis represents actual survival and 95% CIs measured by Kaplan-Meier analysis

The nomogram that integrated all the 10 independent factors for OS in the training cohort to predict the 90-day mortality is shown in Fig. 1. The C-index for the model prediction was 0.76(95% CI: 0.73–0.80)in the training model. The calibration plot for the OS showed an optimal agreement between the prediction by the nomogram and actual observation (Fig. 2). An additional 131 advanced cancer patients with palliative care from January 2015 to October 2015 were independently included to validate the nomogram. The nomogram assigned a score to each patient in the validation cohort, which showed an ideal correlation with the actual survival (C-index 0.75; 95% CI: 0.70–0.80). Furthermore, the external calibration plot for the OS also showed good agreement (Fig. 3). As expected, the DCA yielded a wide range of risk thresholds, at which the clinical net benefits would be obtained from our proposed nomogram (Additional file 1: Figure S2).

## Discussion

The survival prediction for advanced cancer patients in the palliative care unit was a global challenge and could have clinical, administrative, and academic significance. In the above analysis, we identified 10 independent predictors (sex, age, tumor stage, KPS, neutrophils, hemoglobin, LDH, albumin, UA, and cystatin-C) for OS and then developed into an easy-to-use, well-calibrated, and externally valid nomogram to predict the 90-day mortality of advanced cancer patients receiving palliative care. This novel nomogram may be useful for routine clinical practice.

The nomogram is an important statistical model that incorporates multiple risk factors into the complex mathematical calculation with pictorial representations [11]. The nomogram has been well recognized for many cancers for predicting survival, and it has demonstrated superiority over the traditional TNM staging system [8, 15, 16]. Despite an increasing number of studies on survival prediction for advanced cancer patients in the palliative care unit, somewhat well-validated prognostic models, such as the Palliative Performance Scale and the Palliative Prognostic Score, have wide variabilities in clinical application [10, 17]. In addition, estimation based on a multivariable model is now considered more reliable than a single risk factor for estimating risk probabilities [18]. In this scenario, we questioned whether the nomogram might improve our ability to estimate the prognosis of advanced cancer patients.

In this analysis of 247 advanced cancer patients from the palliative care unit, 85.4% of the patients had died within 90 days. When pre-palliative care factors were investigated, we found that female sex, old age, advanced tumor stage, poor KPS, and abnormal laboratory parameters (e.g., neutropenia, hypohemoglobinemia, hypoalbuminemia, hyperuricemia, elevated LDH or cystatin-C) were associated with an increased likelihood of death in the final phase. To identify patients with a dramatically increased risk of early mortality, we constructed and validated a novel easy-to-use nomogram to aid clinical prognostication and facilitate individualized evaluation of advanced cancer patients. This nomogram incorporates 10 independent prognostic parameters that are readily available during pre-palliative care based on the results of the multivariable analysis.

Regarding the individual variables in the nomogram for overall survival, demographic characters, such as female sex or increasing age, were associated with a worse prognosis. It is known that the effects of sex differences on overall survival may vary by different types of cancer. However, female patients may suffer more from psychological distress, leading to a poorer quality of life and survival [19, 20]. Overall survival may show a greater association with elderly patients due to a more advanced stage of cancer, more comorbidities, a poorer performance status, and lower treatment compliance [16, 21]. In addition, we found that tumor stage and KPS were significantly associated with OS for advanced cancer patients in the palliative care unit. This finding is not surprising. Tumor stage, which reflects the underlying tumor biology, has been acknowledged as the main predictor of OS in many cancers [22, 23]. KPS, a universal assessment qualifying the actual level of function and self-care ability of cancer patients, also strongly predicts the probability of death in many cancers [24, 25]. Other prognostic factors identified in our study were abnormalities in laboratory parameters, including neutrophils, hemoglobin, LDH, albumin, UA and cystatin-C. All these laboratory parameters are easily measurable at most centers. Among these abnormal laboratory parameters, neutrophils, LDH, UA, and cystatin-C are all linked to the inflammatory microenvironment, which can significantly impact tumor development and progression [26–29]. Consistent with prior research [30–33], neutropenia, hyperuricemia, elevated LDH and cystatin-C were associated with unfavorable outcomes for advanced cancer patients in the palliative care unit regardless of the cancer origin and other clinical characteristics and biomarkers. Hemoglobin and albumin are two commonly used markers for nutritional status, which sharply decline during the progression of cancer. Numerous studies have demonstrated the correlation of hemoglobin and albumin levels with OS in various cancers [34–36]. The current study results correspond well with previous reports showing that both low pre-palliative care hemoglobin and albumin levels are independent predictors of mortality in advanced cancer patients in the palliative care unit. Based on these significant clinical and biochemical indicators, a novel prognostic nomogram with a high C-index of 0.76 was developed in the training set. The discriminative ability was reproduced in the external validation set with a satisfactory calibration and discrimination of 0.75. The C-index values for the validation set were similar to those for the development set, ensuring reliable performance.

In the palliative care unit, both physicians and patients are consistently faced with decisions - to administer further intensive antitumor therapy or offer patients the best supportive care. Decisions should be balanced with a careful evaluation of financial burden, the patient’s life expectancy and the quality of his or her remaining life. Under this scenario, an accurate prognostic assessment is essential to guarantee appropriate decision-making and management of these patients, minimizing the risk of overtreatment or undertreatment. In the current study, a novel prognostic nomogram was developed from individual prognostic factors that could be easily obtained in routine clinical practice. This nomogram can stratify advanced cancer patients into well-identified death-risk groups and individually predict their clinical course and optimal treatment. In particular, patients with a low risk for mortality could choose a specific antitumor therapy to alleviate symptoms and extend life, while high-risk patients with negligible chances for recovery should undergo further enhanced palliative care. Hopefully, this nomogram will assist clinicians, patients and families in real-world clinical decision-making to improve the individualized care of patients with advanced cancer.

To the best of our knowledge, there are only 3 reports investigating a prognostic nomogram for mixed cancer patients in the palliative care unit. Two were performed in 2009 and 2012 in mixed advanced cancer patients and constructed the nomogram based mainly on clinical signs and symptoms, completely excluding biological markers [37, 38]. The other study was published in 2011 using terminally ill cancer patients and developed a nomogram based mainly on laboratory variables [8]. However, a major limitation of this study arises from the lack of a clinical criterion for the definition of terminally ill cancer patients and a diagnosis that is dependent on each physician’s evaluation. Inevitably, the study included more subjective factors, leading to less accurate results and clinical applicability. Thus, the present study is the first to generate a prognostic nomogram using various patient and laboratory characteristics for advanced cancer patients in the palliative care unit.

Although the novel nomogram achieved excellent performance, certain limitations of our study should be mentioned. First, the study was conducted in a single institution retrospectively with inherent selection bias. Second, the lack of a validation group outside the FUSCC may limit the generalization of our findings. Therefore, prospective and multi-institutional studies are needed prior to full application of the nomogram in daily clinical practice. In addition, we prepared to integrate a web-based electronic predictive tool, which is convenient and user-friendly for both clinicians and patients, in the near future.

## Conclusions

We developed and externally validated a novel prognostic nomogram for advanced cancer patients in the palliative care unit, which may valuable for guiding individual palliative care for advanced cancer patients. Thus, we believe that this novel nomogram merits a detailed analysis using a large cohort for wide applicability in daily clinical practice.

## Additional file

Additional file 1: **Figure S1.** Flow chart of the survival data collection. **Figure S2**. DCA for the nomogram. (DOC 119 kb)

## Data Availability

The datasets used and analyzed during the current study are available from the corresponding author on reasonable request.

## Abbreviations

ALT: Glutamic-pyruvic transaminase
AST: Glutamic-oxalacetic transaminase
CEA: Carcinoembryonic antigen
KPS: Karnofsky performance status score
LDH: Lactate dehydrogenase
OS: Overall survival
UA: Uric acid
