# Supplementary material for: Nomogram-based parameters to predict overall survival in a real-world advanced cancer population undergoing palliative care
Source: BMC Palliat Care. 2019 Jun 5;18:47. doi: 10.1186/s12904-019-0432-7 (PMC6551870; doi:10.1186/s12904-019-0432-7)
Supplement: Supplementary file 1 — Figure S1. Flow chart of the survival data collection. Figure S2. DCA for the nomogram. (DOC 119 kb) [file 12904_2019_432_MOESM1_ESM.doc]

**Supplementary Information**

**Supplementary Methods**

Details of inclusion/exclusion criteria and follow-up procedure are described as follows:

Patients with the following inclusion criteria were enrolled: (1) a hospitalization for palliative care; (2) the presence of various cancers confirmed by histopathology or at least cytology; (3) availability of pretreatment peripheral blood test results from 1–3 days prior to palliative care; and (4) availability of all clinical data. Patients with benign or early stage (I,II) tumors, and those with active infectious disease were excluded from the analysis.

In our study, death was observed in 309 of 378 patients. Survival data were collected mainly by two ways: (1) About one thirds of the patients were dead in our hospital, the survival time was extracted from their medical records; (2) For the rest of the enrolled patients, the survival time was collected via follow-up by trained staff from Clinical Statistics Center of Fudan University Shanghai Cancer Center, which was established to specifically responsible for the work of follow-up in 2009.

If one patient returned for a post-treatment checkup, the required information from either in-patient or out-patient information system would be automatically linked to the follow-up platform. Therefore, his/her follow-up information (recurrence, no-recurrence, metastasis, no metastasis) could be updated by our follow-up workers. If one patient did not returned more than one year after discharged from our hospital, passive follow-up method needs to be used to get the information of his/her current status. The strategy was first to collect those patients (lost of contact for more than one year) into a frame. Then, our follow-up staff would contact the patients in frame via telephone calls. If no one answered the phone, we sent a mail to the correspondence address. If there was still no response, those patients would move to a special database called “Hold file”. If any patient in “Hold file” database paid a visit to our hospital again during the time we were trying to contact him/her, he/she would be moved back to the medical record follow-up platform. If the information of patients could not be get from both active and passive methods, we matched the death information in the vital statistics system of Health Statistics Department of Shanghai Municipal Center for Disease Control & Prevention (CDC). If there was still no information, this patient was defined as “Censor”. The complete flow of our follow-up is illustrated in the following Figure (Figure S1).

Yes

No

No

No

Yes

Yes

Returned to hospital

Follow-up via mail

Update the follow-up information

Patients need to be followed up

Follow-up via Telephone

Get the information

Get the information from in-patient and out-patient records

Censor

“Hold file” database

Match information with CDC

Get the information

**Figure. S1 Flow chart of the survival data collection.**


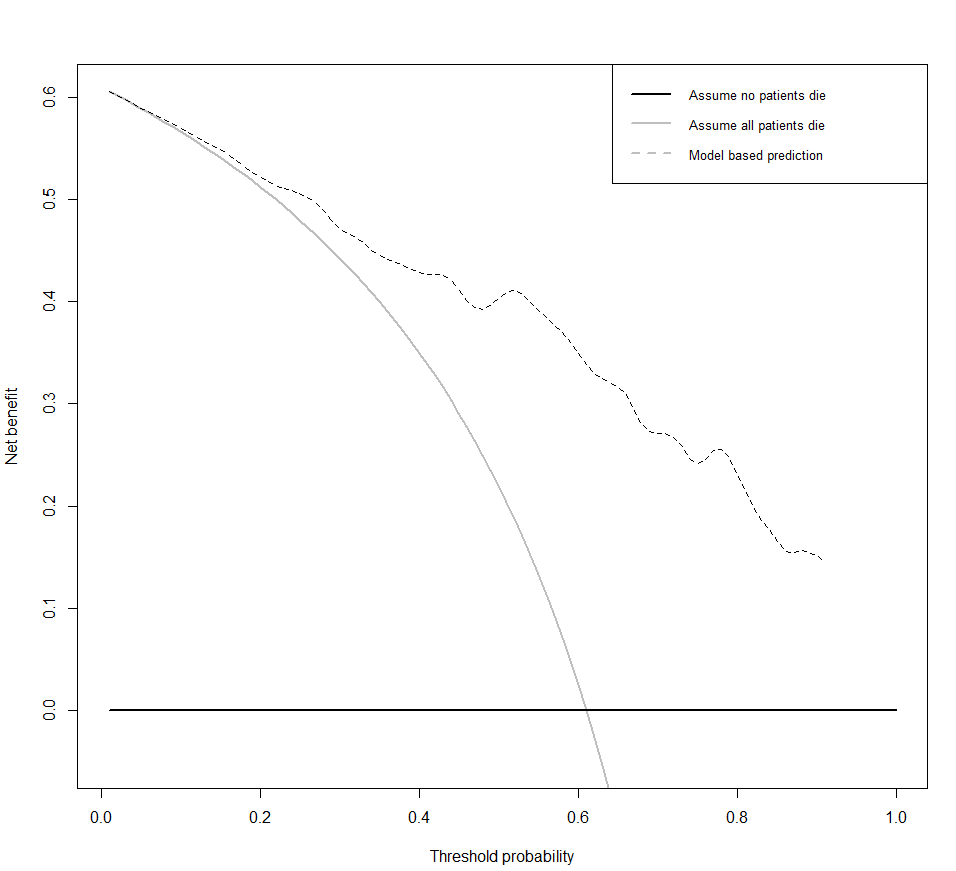


F**igure. S2 DCA for the nomogram.**

The y-axis represents the net benefit. The dashed line represents the nomogram based prediction. The gray solid line represents the hypothesis that all patients would die. The black solid line represents the hypothesis that no would die. The x-axis represents the threshold probability. The decision curve showed that if the threshold probability was between 10% and 95%, then using the nomogram to predict the probability added more benefit than treating either all or no patients would die.
